# Effective Reconstruction of Functional Urethra Promoted With ICG-001 Delivery Using Core-Shell Collagen/Poly(Llactide-co-caprolactone) [P(LLA-CL)] Nanoyarn-Based Scaffold: A Study in Dog Model

**DOI:** 10.3389/fbioe.2020.00774

**Published:** 2020-07-10

**Authors:** Kaile Zhang, Xiaolan Fang, Jingjing Zhu, Ranxing Yang, Ying Wang, Weixin Zhao, Xiumei Mo, Qiang Fu

**Affiliations:** ^1^Department of Urology, Affiliated Sixth People’s Hospital, Shanghai Jiao Tong University, Shanghai, China; ^2^Diagnostic Laboratory, Greenwood Genetic Center, Greenwood, SC, United States; ^3^Biomaterials and Tissue Engineering Laboratory, College of Chemistry, Chemical Engineering and Biotechnology, Donghua University, Shanghai, China; ^4^Wake Forest Institute for Regenerative Medicine, Winston-Salem, NC, United States

**Keywords:** urethral stricture, nanoyarn, Wnt signaling, urethral reconstruction, urethroplasty

## Abstract

Hypospadias and urethral stricture are common urological diseases which seriously affect voiding function and life quality of the patients, yet current clinical treatments often result in unsatisfactory clinical outcome with frequent complications. *In vitro* experiments confirmed that ICG-001 (a well-established Wnt signaling inhibitor) could effectively suppress fibroblast proliferation and fibrotic protein expression. In this study, we applied a novel drug-delivering nanoyarn scaffold in urethroplasty in dog model, which continuously delivers ICG-001 during tissue reconstruction, and could effectively promote urethral recovery and resume fully functional urethra within 12 weeks. Such attempts are essential to the development of regenerative medicine for urological disorders and for broader clinical applications in human patients.

## Introduction

Hypospadias and urethral stricture are common urological diseases which seriously affect voiding function and life quality of the patients. Current clinical treatment for such urethral defects are mainly based on transplantation of autologous tissues (e.g., penile flap or buccal mucosal graft), which is frequently accompanied with severe side effects (such as local injuries) and is often limited by the extremely low tissue amount from penile and oral mucosa. In addition, the efficiency of urethral repair might be compromised by insufficient wound healing or stricture formation in the lumen. There is an urgent need for novel biomaterials and clinical methods to facilitate the reconstruction of functional urethra from such urethral defects, and simultaneously promote wound healing and prevent fibrosis/scar formation for a satisfactory tissue recovery.

The rapid development of biomaterials brought in many trials of tissue-engineered urethra in preclinical and clinical studies, yet the successful rate varies, and the efficacy is often disappointing, especially in the scaffold without pre-loaded cells (Raya-Rivera et al., 2011; Li et al., 2013; Xie et al., 2013, 2014; Ramsay et al., 2016; Atala et al., 2017; Kajbafzadeh et al., 2017; Zhou et al., 2017). One possibility is that the small pore size of the experimental biomaterial might hinder the infiltration of local cells from the native tissue into scaffold. Another possibility is that the fibrosis related signaling [e.g., TGF-beta, connective tissue growth factor (CTGF/CCN2), platelet-derived growth factor (PDGF), interleukin 4 (IL-4), etc.] pathways are activated during the urethral reconstruction process, which promotes fibrosis and recurrence of urethral stricture (Sangkum et al., 2015). Effective inhibition of such signaling pathways are critical for post-injury tissue regeneration, particularly in tissue-engineered urethra models which human cells are pre-planted to bridge the novel urethra to the native urethra edges (Raya-Rivera et al., 2011; Guo et al., 2016). Although it has been reported that some stem cells might possess the property of inhibiting inflammation and fibrosis, previously applications of engineered human cells confronted a number of drawbacks and limitations in urethral reconstruction and stricture occurrence (Versteegden et al., 2017). Therefore, it has been a challenge to use the combination of biomaterial and preloaded human cells in the treatment of urethral defects *in vivo*, and it remains even more difficult to get translated from bench side to bedside.

**FIGURE 1:**
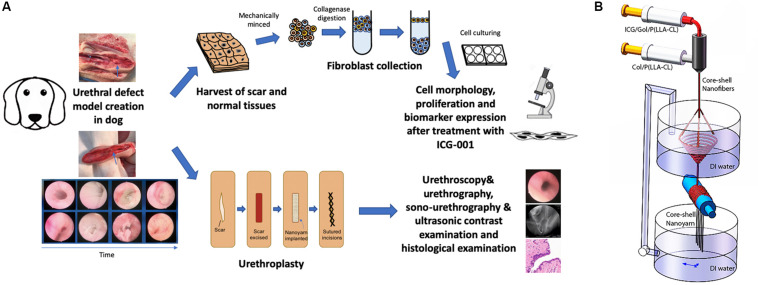
Overview of key components in current study. **(A)** The flowchart of study design. **(B)** Fabrication process of ICG-001 delivered nanoyarn. The core-shell electrospinning system (top) contains two syringes containing ICG-001/Col/P(LLA-Cl) and Col/P(LLA-Cl), respectively. The dynamic liquid system (bottom) is integrated with core-shell electro-spinning system.

In this study, we utilized a novel bioengineering method and produced a functional electrospun collagen/poly(L-lactide-co-caprolactone) [P(LLA-CL)] nanoyarn scaffold (Figure 1B) to improve urethroplasty efficiency. Nanoyarn has been verified to increase pore size comparing to traditional conjugated nanofibrous scaffold *in vitro* (Guo et al., 2016) and is expected to serve as a promising biomaterial for tissue reconstruction. A co-axial electrospinning system and a dynamic liquid system were integrated to deliver the Wnt pathway inhibitor ICG-001 and the drug release efficacy was evaluated *in vitro* (Guo et al., 2016). ICG-001 specifically binds to CREB-binding protein (CBP) and has been used widely as an antagonizer of Wnt/β-catenin-mediated transcription. We applied optimized concentration of ICG-001 in dog model and evaluated the urethroplasty outcome based on urethroscopy, urethrography, sono-urethrography and histology analysis (Figure 1A).

## Materials and Methods

### Biomaterial for Nanoyarn Production

Poly(L-lactide-co-caprolactone) [P(LLA-CL)] (LA:CL = 50:50, *MW* = 300,000) was purchased from Daigang bioengineering Co., Ltd. (Jinan, China). Type I collagen was purchased from Ming-Rang BioTech Co., Ltd. (Sichuan, China). 2, 2, 2-trifluoroethanol was purchased from Fine Chemicals (Shanghai, China). ICG-001 was purchased from Selleck Chemicals (Shanghai, China).

### Core-Shell ICG-001-Delivering Nanoyarn Fabrication

The fabrication of nanoyarn was reported previously using a co-axial electrospinning device (Donghua University, Shanghai) (Zhang et al., 2016; Figure 1B). Briefly, a hole (8 mm in diameter) was created in a basin, which allows the flow of water to form a water vortex. A pump was employed to recycle water back to maintain the water level after the water was drained through the hole into a tank below the basin. Electrospun nanofibers were generated and deposited on the water surface; then, the nanofibers were twisted into a bundle of nanoyarn in the water vortex and collected by a rotating mandrel (60 r/min) to form a nanoyarn scaffold. Nanofibrous scaffold fabricated with conjugated electrospinning technique was set as control group to compare the morphology and mechanical property with nanoyarn. For the construction of Collagen/P(LLA-CL) scaffolds, the solution of the core layer was 1 g collagen/P(LLA-CL) dissolved in 2, 2, 2-trifluoroethanol. Then it was mixed with 0.1, 0.5, 1, 2, and 4 mg ICG-001, respectively, in 60 μL DMSO solution and injected at a rate of 0.2 ml/h. The solution of the shell layer was 1g Collagen/P(LLA-CL) dissolved in 2, 2, 2-trifluoroethanol and fed at 0.8 ml/h. During the process of scaffold fabrication, room temperature was maintained at 22–25°C, and the relative humidity at 40–50%. A dynamic liquid system was used to collect the nanofibers to fabricate the ICG-001 delivering nanoyarn. The distance between the sprayer tip and the receiving water level was set to 15 cm and the positive voltage was 18 kV.

### Scanning Electron Microscopy

Scanning electron microscope (SEM, Hitachi TM-100, Tokyo, Japan) was used to observe morphology of the scaffolds. Specimens were punched into 1.2 cm-diameter disks and cryopreserved at −80° for 2 h, then freeze-dried overnight and preserved in a vacuum container. Fibroblasts were seeded on the nanoyarn and conjugated nanofibrous scaffold specimens in the 24 wells culture dish for 3 days. The specimens with or without cells were imaged under SEM on first day and third day. The angle distribution was measured from 100 yarns in the SEM images.

### Mechanical Property Test

Universal materials tester (H5K-S, Hounsfield, United Kingdom) was used to evaluate the tensile strength of the drug delivering nanoyarn. The conjugated electrospun nanofibrous scaffold and bladder acellular matrix graft (BAMG) were used as the control material. All the scaffold samples were prepared as longitudinal strips (20 mm in length and 10 mm in width). Each sample of scaffolds was fixed onto the clamps and pulled at 5 mm/min crosshead speed until rupture. The stress and strain data in the process were recorded.

### Fourier-Transform Infrared Spectroscopy

The chemical components of ICG-001 delivering nanoyarn and its control scaffolds were, respectively, characterized by Fourier transform infrared spectrum (FTIR, Thermo Electro AVATAR 380, United States).

### Fibroblasts Isolation

The animal protocol (SYXK 2011-0128) was approved by the Animal Ethics Committee of Shanghai Sixth People’s Hospital, Shanghai, China. The project identification code is 14JC1492100, which was approved on September 1, 2014 by Science and Technology Commission of Shanghai, China. All the animal experiments were performed in accordance with the guidelines for animal care equivalent to the NIH Guide for Care and Use of Laboratory Animals. All the beagle dogs were with average body weight (BW) ∼20kg and were provided by Shanghai Academy of Agricultural Sciences.

Skin biopsies and fibroblasts harvest were performed to evaluate the biocompatibility and fibrosis inhibiting effect of ICG-001 delivering nanoyarn. Three beagle dogs were used. Each dog was pretreated with 15 mg/kg Ketamine, 2–3 mg/kg xylazine and 0.75 mg/kg acepromazine intramuscularly. Then they were anesthetized and maintained with 2% isoflurane. A small laparotomy excision was made above the pubic symphysis. A biopsy of skin specimen with 2 cm × 2 cm was excised from the skin of abdomen wall, then the defect was closed with 3-0 polyglactin sutures in two layers. The specimen was processed in a sterile condition. It was washed with PBS with 100 IU/mL penicillin and 100 μg/mL streptomycin. The fibroblasts were isolated, sorted and stored as previously reported (Guo et al., 2016), and were used in *in vitro* experiments in this study. The animals were euthanized after tissue collection.

### Preparation of Fibroblasts Treated With ICG-001 Releasing Solution

To prepare the ICG-001 releasing solution, the ICG-001 delivering core-shell nanoyarn with different amount of ICG-001 was sterilized with ultraviolet for 2 h. To collect the ICG-001 medium, 2 mL of complete culture medium was used to immerse 120 mg ICG-001 delivering nanoyarn to get ICG-001 solution for 24 h. Six ICG-001 released media were prepared with nanoyarn with different concentrations of ICG-001 [0.1, 0.5, 1, 2, 4 mg and negative control (complete culture medium without ICG-001)].

To prepare the fibroblast treated with ICG-001 releasing solution, ten thousand fibroblasts were transferred to each well of 4-well chamber slides and cultured overnight. Five groups were set in the study by adding different IGC-001 solutions from the previous step. The fibroblasts were cultured for 3 days before they were used in further experiments.

### Cell Morphology and Proliferation Assays

After culturing with ICG-001 containing culture medium for two days, the cells’ morphology was observed under microscope, and cell proliferation was evaluated by the 3-(4,5-dimethylthiazol-2-yl)-2,5-diphenyltetrazolium bromide (MTT, Product #5655, SigmaAldrich, St. Louis, United States) assay at day 2 and 6 after seeding according to the protocol. After incubation at 37°C for three hours, the medium was transferred into 96-well plate. The data were read by enzyme-labeled instrument (Multiskan MK3, Thermo Fisher Scientific, Waltham, United States) at 492 nm to measure the absorbance of the solution.

### Western Blot

Western blotting analysis was conducted to analyze the relative expression level of collagen type 1 and 3 in fibroblasts treated with culture medium released from the ICG-001 delivering nanoyarn as previously reported (Guo et al., 2016). The results were normalized using the expression of β-actin (Anti-beta Actin antibody, Cat# ab8226, Abcam, Cambridge, MA, United States). Mouse-anti-Collagen I and anti-Collagen III were purchased from Sigma-Aldrich, St. Louis, MO, United States (Cat# C2456 and Cat# C7805). Samples were incubated with primary antibodies at 4°C overnight and subsequently with HRP-conjugated goat anti-mouse secondary antibody (Cat# ab97023, Abcam, Cambridge, MA, United States) for one hour at room temperature. Anti-GAPDH antibody (Cat# ab9484, Abcam, Cambridge, MA, United States) was used as a protein loading control. The results were quantified using Quantity One software (version 4.5.2) and expression was normalized by comparing to GAPDH. The expression levels were compared using one-way ANOVA by Prism8 (GraphPad).

### Urethroplasty and Postoperative Examinations in Beagle Dog

**FIGURE 2:**
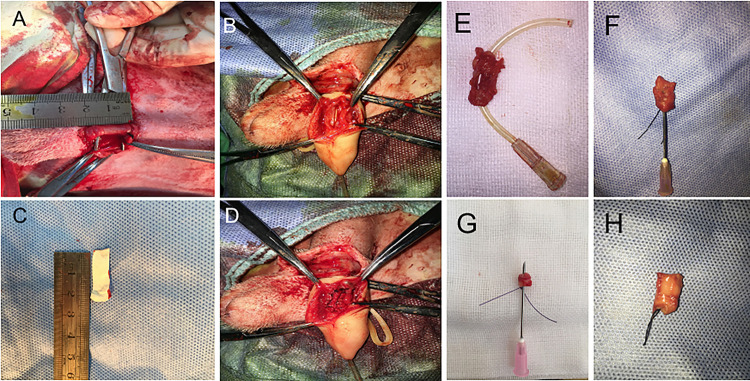
The surgery process of urethral defect model creation and urethroplasty with biomaterial. **(A)** Dog urethra (diameter = 2 cm) was exposed and measured. **(B)** F8 catheter was inserted from the penile tip and kept in urethra, and the urethral wall was opened to 2 cm in length with a pair of scissors. **(C)** 2 cm × 0.5 cm ICG-001 delivering nanoyarn was prepared. **(D)** The ICG-001 delivered nanoyarn was implanted into the urethral defect area. **(E)** The fistula (in the gray dotted rectangle) formed in the middle of the repaired segment with conjugated scaffold. **(F)** Stricture formed in the urethra repaired by nanoyarn (the needle cannot pass through). **(G)** Unobstructed urethra was repaired with ICG-001 delivered nanoyarn (the needle could pass through). **(H)** A healthy urethra without repair was shown as a control.

For urethral defects and follow-up urethroplasty, ten beagle dogs were used. The animals were randomly divided into 3 groups. Dogs in group 1 (*n* = 2) were treated with conjugated nanofibrous scaffold. Group 2 (*n* = 4) were treated with nanoyarn without ICG-001. Group 3 (*n* = 4) were treated with ICG-001 delivering nanoyarn. After general anesthesia (as described in Fibroblasts Isolation section), Foley F8 silicone catheters (Suzhou, Jiangsu, China) were inserted into the urethras (Figures 2A,B). Briefly, the skin approximately 3 cm from the external urethral orifice was sectioned, and the urethra was dissected from the corpus cavernosum. Ventral urethral defects (mean length of 2.0 cm and width of 0.8 cm) were created in the urethra of penile part of dogs. The scaffolds (length of 2 cm and width of 1 cm) were sutured to the defect with 6–0 absorbable polyglactin sutures (Figures 2C–H). The 8F silicone catheter was left in the urethra and fixed with 4–0 suture at the dog’s gland, and with 6–0 absorbable sutures for 14 days postoperatively. The animals were observed twice daily before catheters were removed. In case the catheter was removed by the animal, another new catheter would be reinserted after anesthesia. The urethroscopy was used to observe the recovery process of the urethra. Photos of the urethral lumen were captured at 6 and 12 weeks post-surgery. Retrograde urethrograms were performed for the animals in 3 groups to assess urethral caliber under anesthesia at 6 and 12 weeks post-surgery. The contrast solution was injected into the urethra lumen to observe any leakage and stricture. Also, the dogs were undertaken the urethra contrast-enhance ultrasound test to check the condition of scar in the urethra at 6 and 12 weeks post-surgery. The bubble contrast solution was used to enhance the resolution. The animals were euthanized after urethroscopy and retrograde urethrograms at 12-week period. Urethral tissues were harvested for the following staining experiments.

### Histology and Immunohistochemistry

The urethra specimens were rinsed with PBS and fixed in 4% paraformaldehyde for 15 min at room temperature followed by dehydration and paraffin embedding. Hematoxylin-eosin (H&E) and Masson staining were performed according to the protocol to evaluate vascularization and collagen distribution. For immunohistochemistry, the slides were processed and cryosectioned at 12-μm thickness using a cryostat (Leica CM1950). Immunohistochemical staining were performed using monoclonal antibodies against the SMCs (α-actin; Santa Cruz, Dallas, TX, United States), AE1/AE3 and CD31 (Cat#41-9003-82, and Cat#CF504773, Thermo Fisher Scientific, Waltham, United States). Then, the specimens on slides were treated with Biotin-Streptavidin (ABC) and DAB in Biotin-Streptavidin (ABC) IHC detection kits (Cat# ab64264, Abcam, Cambridge, MA, United States). The tissue sections were counter-stained with hematoxylin for the nuclei.

## Results

### Morphology of Drug-Delivering Nanoyarn With Pre-loaded Fibroblasts

**FIGURE 3:**
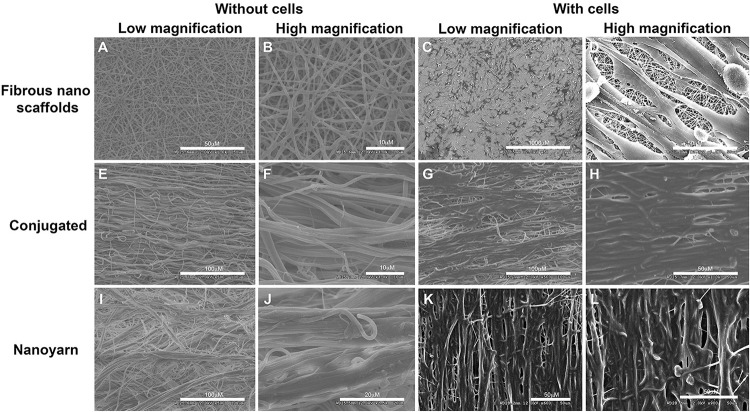
Physical characteristics of different nano scaffolds under SEM. Fibrous nano scaffolds **(A–D)**, conjugated nanofibrous scaffold **(E–H)** and nanoyarn **(I–L)** were observed at different magnifications with or without preloaded fibroblasts. Scale bars are indicated in each figure.

The thickness was measured for fibrous nano scaffolds (0.75 ± 0.16 mm, mean ± std), conjugated nano scaffolds (0.90 ± 0.21 mm), nanoyarn (0.93 ± 0.19 mm) and ICG-001 delivering nanoyarn (0.92 ± 0.14 mm), as well as the diameter (conjugated nano scaffolds (1.51 ± 0.351 μm), nanoyarn (2.447 ± 0.408 μm) and ICG-001 delivering nanoyarn (2.547 ± 0.508 μm), as previously reported (Guo et al., 2016). The thickness of core layer was 53 ± 15 nm, and the shell layer was 226 ± 27 nm. The water contact angle was measured as previously reported (fibrous nano scaffolds, 120.97 ± 6.13°; ICG-001 delivering nanoyarn, 87.9 ± 5.78°) (Guo et al., 2016). To confirm the physical properties of nano biomaterials for fibroblast growth, we observed the morphology of nanoyarn and the conjugated nanofibrous scaffold under SEM. For both of them, the direction of fibers was aligned. The angle distribution of the nanoyarn was 8.3 ± 4.5 degrees (mean ± std). Compared with conjugated nanofibrous scaffold (Figures 3A,B), the nanofibers in nanoyarn (Figures 3E,F) were twisted into thick yarns with more fibers, indicating a higher flexibility for the engineered tissue. This was confirmed in the following animal experiments, as nanoyarn was very convenient to handle and easy to suture during the surgery, and closely mimicking the native tissue to resume urethral function/plasticity. The pore size exhibited in nanoyarn was larger than conjugated nanofibrous scaffold (Figure 3), which would allow efficient cell infiltration from neighboring tissues. Compared with cells on conjugated nanofibrous scaffold (Figures 3C,D), in the cells-laden nanoyarn the cells grew along the yarns with abundant extracellular matrix (ECM) (Figures 3G,H). The quantitative data of the pore size, porosity and cell infiltration of nanoyarn, conjugated nano scaffold and nanofibrous scaffold was reported previously (Guo et al., 2016).

**FIGURE 4:**
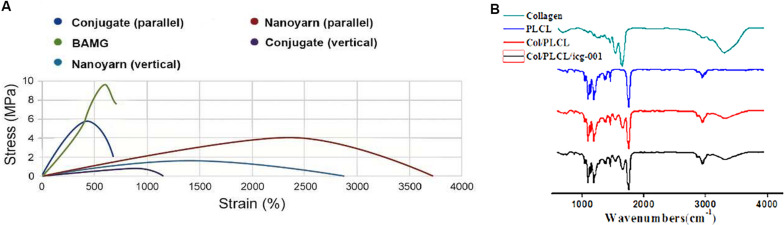
Mechanical properties of different nano scaffolds. **(A)** The tensile strength of nanoyarn, conjugated scaffold and BAMG. **(B)** FTIR for different polymers in PLCL, control nanoyarn and drug (ICG-001) delivering nanoyarn.

To optimize the mechanical characteristics of the nano biomaterials for *in vivo* experiments, we modified the setting of the parameters in previous experiments (Zhang et al., 2016), and evaluated the updated mechanical properties of various nano scaffolds (Figure 4A and Table 1). Comparing to non-woven nanofibrous scaffolds (BAMG) and conjugated nano scaffolds, nanoyarn showed lower maximum tensile strength and Young’s modulus, higher elongation at break and higher elasticity in vertical and parallel directions (Figure 4A and Table 1), indicating better plasticity and strength. The properties of the present nanoyarn is more suitable for suture and manipulation in the dog model operation. In addition, we previously evaluated the release efficacy and time range of ICG-001 from three different nano scaffolds *in vitro* (Supplementary Figure 1; Guo et al., 2016). The ICG-001 delivering nanoyarn showed similar polymer constitution comparing to control nanoyarn [collagen conjugated (Col/PLCL) or non-conjugated (PLCL)] (Figure 4B), suggestion that the load of ICG-001 in the whole nanoyarn doesn’t affect its physical composition, which is expected.

**TABLE 1:** The stress, strain and Young’s modulus of various biomaterials (BAMG, conjugated scaffolds and nanoyarn).

	**Stress (MPa)**	**Strain (%)**	**Young’s modulus (Mpa)**
BAMG	9.52 ± 1.32	62735	1.52 ± 0.13
Conjugate (parallel)	5.85 ± 0.37	41554	1.43 ± 0.21
Conjugate (vertical)	0.46 ± 0.13	83668	0.53 ± 0.09
Nanoyarn (parallel)	4.13 ± 0.29	2385216	0.16 ± 0.03
Nanoyarn (vertical)	1.87 ± 0.13	1423153	0.13 ± 0.02

### Released Medium From Nanoyarn Pretreated With 4 mg/ml ICG-001 Could Effectively Inhibit Fibroblast Proliferation and Suppress Protein Expression of Fibrosis Associated Proteins *in vitro*

**FIGURE 5:**
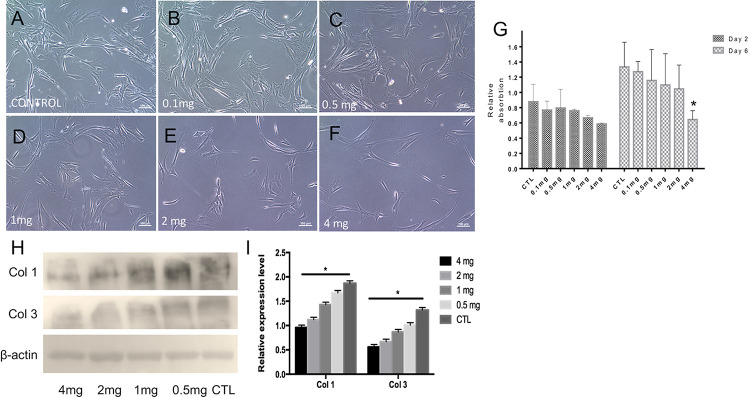
Cell morphology, proliferation and collagen expression based on treatment with ICG-001 at various concentrations. **(A–F)** Fibroblasts incubated with released media from cells treated with different concentrations (0.1, 0.5, 1, 2, and 4 mg) of ICG-001 were observed under light microscope. Control (CTL) is not treated with ICG-001. **(G)** Cell proliferation was evaluated by MTT assay. **(H,I)** Western blotting and relative expression quantification of fibrosis related proteins [Collagen type I (Col 1), Collagen type III (Col 3)] were evaluated. *indicates significant difference (*p* < 0.05, comparing to control).

To determine the optimal dosage of ICG-001 for effective inhibition of fibroblast proliferation and suppressed expression of fibrotic factors (e.g., Collagen I, Collagen III, etc.), we performed biocompatibility and anti-fibrosis assays. The released media from nanoyarn with 2 or 4 mg ICG-001 resulted in an obvious inhibition of cell growth in fibroblasts by morphological observation (Figures 5A–F). The cells maintained normal proliferation in less than 1 mg/ml released medium (Figures 5A–D). The cell proliferation inhibition effect was validated by MTS assay, which confirmed a significant decrease of proliferation with the 4 mg-ICG-001 medium at day 6 (Figure 5G). Significant decrease of Collagen I and III expression was also confirmed with the 4mg-ICG-001 medium (Figures 5H,I).

### ICG-001 Delivering Nanoyarn Could Effectively Promote Urethral Reconstruction and Resume Fully Functional Urethra *in vivo*

**FIGURE 6:**
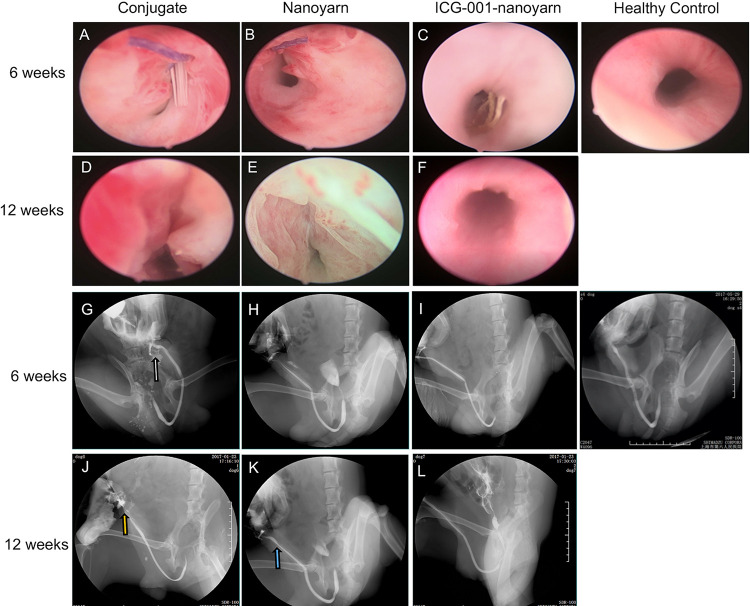
Urethroscopy and urethrography examinations for urethras. Urethras repaired with conjugated nanofibrous scaffold **(A,D,G,J)**, nanoyarn **(B,E,H,K)** and ICG-001-delivering nanoyarn **(C,F,I,L)** at 6- and 12-week post-surgery. White and yellow arrow indicate the location of the fistulas. Blue arrow indicates the location of the urethral stricture.

After urethral defects were introduced into the dog models, urethroplasty was performed by implanting various biomaterials into the urethral defects with running suture (Figure 2). The nanoyarn with or without ICG-001 was very convenient to handle and suture, while the conjugated scaffold is pretty fragile. The recovery condition of urethral lumen was checked at 6 and 12 weeks post-surgery. Generally, the 4–0 absorbable Vicryl was still visible in the lumen after 6 weeks but disappeared at 12 weeks (Figures 6A–F). In the urethras repaired with conjugated nano scaffold, obvious fistula at the segment of urethroplasty was revealed (Figures 6A,G). A soft needle could be seen in the inner lumen of urethra when it was inserted from the outside of the fistula at the penile (Figure 6A). After 12 weeks, the fistulas remained in the urethras (Figure 6D) and was also detectable in the urethrography examination (Figure 6J). In the urethras repaired with nanoyarn, majority of urethras were kept unobstructed (Figures 6B,H), although urethral strictures were observed at 12 weeks post-surgery (Figure 6K), as the lumen became very narrow, and the urothelium looked pale, indicating fibrosis associated scar formation (Figure 6E). In the urethras repaired by ICG-001 delivering nanoyarn, unobstructed urethras were revealed at 6 and 12 weeks post-surgery, with no sign of urethral stricture or scar formation (Figures 6C,F,I,L).

**FIGURE 7:**
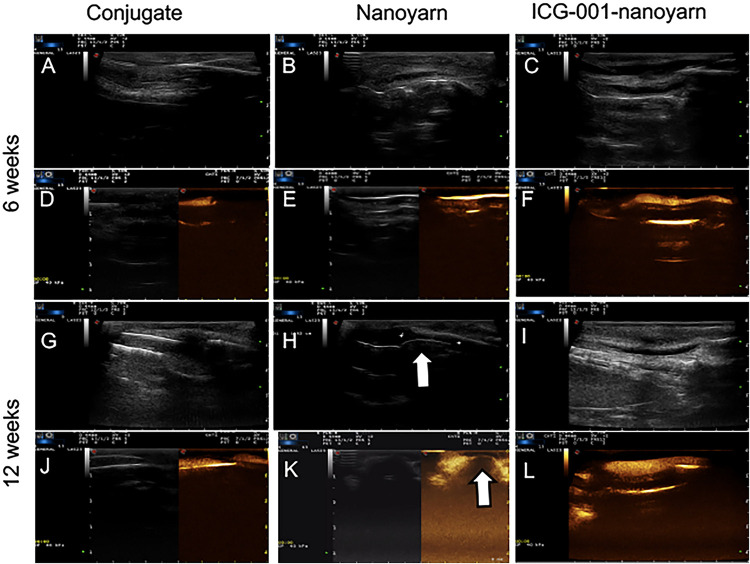
Sonourethrography and ultrasonic contrast examinations for the urethras repaired with conjugated nanofibrous scaffold, nanoyarn and ICG-001-delivering nanoyarn. **(A–C,G–I)** Sonourethrography examination results. **(D–F,J–L)** Ultrasonic contrast examination results. The white arrows indicated the location of scar formation in the urethral lumen.

To further validate those findings, we performed sonourethrography to test the efficacy of the biomaterials for urethroplasty and evaluate the tissue and scar formation in the lumen. 6 and 12 weeks post-surgery, ultrasonic contrast agent was injected into the orifice of the urethra repaired with the conjugated scaffold, but the lumen could not be filled due to the existence of fistula (Figures 7A,D,G,J). In urethra repaired with nanoyarn, the lumen was unobstructed with fluent flow of ultrasonic contrast agent at 6 weeks post-surgery (Figures 7B,E), yet hypertrophy was observed in the lumen which obstructed the flow of contrast agent at 12 weeks post-surgery (Figures 7H,K), likely causing urethral stricture. In the urethras repaired by ICG-001 delivering nanoyarn, the lumen kept unobstructed and wide at 6 weeks (Figures 7C,F) and 12 weeks post-surgery (Figures 7I,L). The overall successful rate of urethroplasty was summarized in Table 2.

**TABLE 2:** The successful rate of urethroplasty with various biomaterials.

	**Conjugated scaffold (*n* = 4)**			**Nanoyarn (*n* = 6)**			**ICG-nanoyarn (*n* = 6)**		
	**Leakage**	**Stricture**	**Success**	**Leakage**	**Stricture**	**Success**	**Leakage**	**Stricture**	**Success**
6 weeks	4	4	0	1	0	5	1	0	5
12 weeks	4	4	0	1	2	3	1	0	5
Success rate		0%			50%			83%	

**FIGURE 8:**
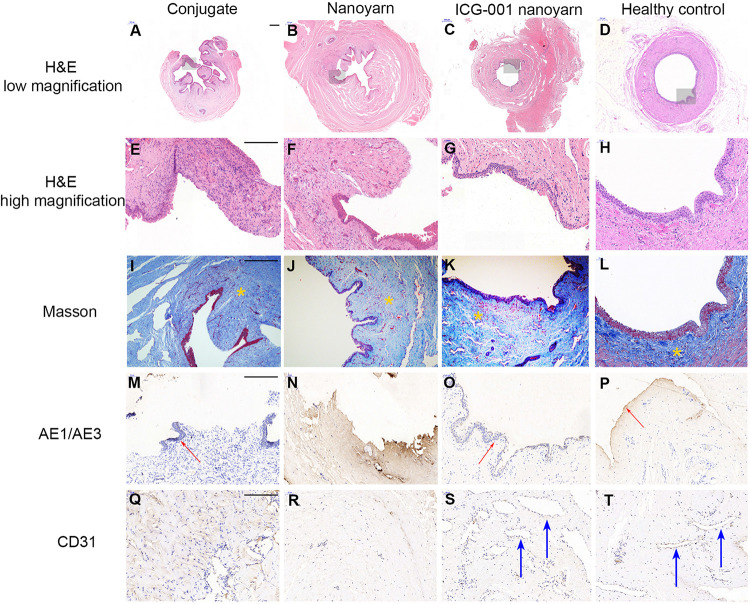
Histological examination of urethras repaired with conjugated nanofibrous scaffold, nanoyarn and ICG-001-delivering nanoyarn. Urethras repaired by various nano scaffolds (12 weeks post urethroplasty) were analyzed based on H&E **(A–H)**, Mansson **(I–L)**, AE1/AE3 **(M–P)** and CD31 **(Q–T)** staining. Yellow asterisks indicate ECM in **(I–L)**. Note the deposited ECM in **(I,J)**, and the well-aligned ECM in **(K,L)**. Red arrows indicate the epithelium in **(M,O,P)**. Blue arrows indicate vessels in **(S,T)**. Scale bar is 200 μm.

To further evaluate the tissue reconstruction at cellular level, we performed immunohistological analysis. The urethras repaired by conjugate nano scaffolds were destroyed by serious fistula and inflammation associated with infection (Figures 8A,E,I,M,Q). In the urethras repaired by nanoyarn, the lumen surface formed discontinued epithelial layer (Figures 8B,F). The tissue showed a large amount of collagen (Figure 8J) and the CD31 expression was low (Figure 8R). In contrast, in the urethras repaired by ICG-001 delivering nanoyarn, the epithelial cells developed regular and continuous epithelium (Figures 8C,G), which is fairly similar to healthy control (Figures 8D,H). The tissue in the submucosa developed less collagen in the Masson image (Figure 8K) and more vessels were formed (Figure 8S).

## Discussion

The muscular anatomy of lower urinary tract, including the surrounding organs, were compared between human and male dogs, and histological analysis confirmed the anatomical resemblance between the two, especially in musculus detrusor vesicae, membranous urethra, prostate, etc. (Stolzenburg et al., 2002). Both male human and canine lower urinary tracts share similar components (urethra, urinary bladder and prostate), although the canines don’t have seminal vesicles or Cowper’s glands. Anatomically, the course of the second dorsal longitudinal urethral muscle system is similar in humans and dogs (Stolzenburg et al., 2002). However, the extension of the ventral longitudinal musculature is restricted to the region caudal of the prostate and it is more strongly developed in dog (Stolzenburg et al., 2002). The similarities and differences are taken into careful consideration during the design of this study. In preparation of the *in vivo* experiments, we have observed the histology of human urethra, which is similar to the canine urethra. The urethral lumen was surrounded by epithelium ECM, vessels and some muscles in the outer layer (data not shown). The thickness is also similar, which is around 1 mm. The observation is consistent with the literature, and it supports that dog model is feasible to test the nano materials for urethral reconstruction and clinical application.

In contrast to traditional electrospinning with simple receiving platform, dynamic liquid system was used to fabricate electrospun scaffold with larger pore size. Core-shell coaxial electrospinning system is popular for producing drug delivered scaffold. In present study, we combined the dynamic liquid system and core-shell electrospinning system together, and discovered that it could be utilized to fabricate a novel electrospun nanoyarn delivering certain kinds of bioactive agents with more stable release. ICG-001 is a Wnt signaling pathway inhibitor, which was reported as an adequate choice for antifibrosis. Thus, to facilitate a biological repair of urethra with less fibrosis and better vascularization and epithelial recovery, ICG-001 was delivered by the nanoyarn. The cells could expand well on the nanoyarn with an aligned growth style, which is necessary to mimic the native collagen fiber of biology.

Compared with BAMG and conjugated scaffold, the nanoyarn is a more elastic material with high strain rate. ICG-001 was delivered by the nano fiber, and the conditioned medium released from ICG-001-nanoyarn showed the effect of inhibition of fibroblasts proliferation and suppression of fibrosis. After the urethral repair using various materials, urethroscopy, urethrographyh and ultrasound showed the best outcome derived from ICG-001 delivering nanoyarn with less leakage and urethral stricture rate. The histology demonstrated that the ICG-001 delivering nanoyarn is not only beneficial for the regeneration of epithelium and vessels, but also for the recovering of normal ECM of urethral by preventing abnormal deposition.

Ideal biomaterial should mimic natural ECM to allow cell adhesion, proliferation and differentiation in biological and architectural features, so as to facilitate tissue formation *in vivo* (Wu et al., 2014). Electrospinning is an efficient and frequently used technique to fabricate scaffolds for regenerative medicine (Barnes et al., 2007). However, due to its nature of layer-by-layer deposition of the nanofibers, traditional electrospun fibers have relatively small pore size which might inhibit cell filtration and hinder regenerative tissue formation (Sill and von Recum, 2008). Thus, how to generate nano scaffolds with 3D microstructures with ideal pore size remains a challenge and a unanswered question in tissue engineering field (Wu et al., 2014; Khorshidi et al., 2016). The previous attempts to solve this problem involved usage of salt leaching, ice crystal formation, sacrificial nanofibers and increasing the fiber diameter etc., which often affect the mechanical property and inhibit the cell growth of fibroblasts due to increased toxicity (Baker et al., 2009). In this study, we improved the traditional non-woven nano fibrous scaffolds and conjugated nano scaffolds, and utilized a novel electrospinning, dynamic liquid system to fabricate the nanoyarn, which has large pore size and 3D microstructures biomaterial through the natural water flow vortex without using any poisonous chemicals. In addition, collagens and P(LLA-CL) were combined to obtain good biocompatibility and mechanical strength. The mechanical property of nanoyarn allowed convenient usage in urethroplasty, and that could be adjusted by shifting the thickness and the density of deposited yarns to use in different types of soft tissues.

How to decrease the drug toxicity and increase drug delivery efficiency is another challenge. Compared with blending drug with the polymer materials directly, the core-shell co-axial electrospinning could decrease the burst release and protect the drug activity in the process of fabrication (Liao et al., 2009; Qian et al., 2014). *In vitro* experiments confirmed that nanoyarn could prevent the burst release in fibrous nano scaffolds, and improve the steady release of ICG-001 in conjugated nano scaffolds (Guo et al., 2016).

TEM of the nanoyarn with ICG-001 was performed, and the data were published in our previous *in vitro* study (Guo et al., 2016), while the present manuscript was more focused on the *in vivo* function of nanoyarn for the urethra reconstruction using dog model. The fibroblasts seeded on the ICG-001 delivering nanoyarn and conjugated nanofibrous scaffold could proliferate well (Figure 3; Guo et al., 2016). In the cell proliferation analysis, we found that the inhibition of fibroblast proliferation and block of collagen deposition showed a dose-dependent pattern. However, the concentration of ICG-001 is not the larger the better. The fibroblasts showed good tolerance to 2mg/ml (and less) ICG-001 in nanoyarn (Figure 5), which was used as the optimized dosage for *in vivo* experiment.

After the urethroplasty with three types of biomaterials (conjugated nano scaffold, control nanoyarn and drug-delivering nanoyarn) in dog models, we evaluated the successful rate based on urethral leakage and recurrent stricture. In the urethra repaired with conjugated scaffold, all the animals had leakage and stricture, probably due to the small pore size and low porosity, and unfavorable mechanical features (Table 2 and Figures 6A,D,G,J, 7A,D,G,J). The regenerated tissue was thin and fragile, and fistula and necrosis occurred. In the urethra repaired by the control nanoyarn, we observed a fair efficacy at six-week (Table 2 and Figures 6B,H, 7B,E). However, at 12-week, the scar in the urethra became serious and led to urethral stricture in two dogs (Figures 6E,K, 7H,K). In the urethra repaired by drug-delivering nanoyarn, we observed the highest overall success rate at 6 and 12 weeks post-surgery. With degradation of the biomaterials and efficient growth of local native cells, little biomaterial could be observed by urethroscopy at six weeks (Figure 6C). This is likely a result of gradual release of ICG-001 from nanoyarn, which suppressed the fibrosis of local cells near the urethral defect during the healing process. The recurrent stricture was completely suppressed in the urethra repaired by drug-delivering nanoyarn and was maintained at 12-week (Figures 6F,L, 7I,L). Urethral leakage was controlled at very low rate for 12 weeks (Table 2). Overall, the drug-delivering nanoyarn showed the most effective clinical advantage in treating the urethral defects and could serve as a promising method to cure human patients who suffer from urethral complications.

For the histology of the urethra repaired with scaffolds (Figure 8), three kinds of scaffolds showed various outcomes. H&E stainning showed a gross condition of the repaired position. Majority of urethra repaired with conjugated scaffold possessed a fistula, and the local tissue is often with inflamation. The nanoyarn without ICG-001 results in a repair with fibrosis, and the epithelial layer could not get a complete regeneration. There was little or trace expression of AE1/AE3. The CD 31 stainning for vessels also showed a low expression in conjugated scaffold repaired urethra and nanoyarn without ICG-001. The CD 31 revealed large lumens of vessels in the urethra repaired with ICG-001 nanoyarn, indicating the tissue formation with more abundant blood supply. The masson stainning is responsible to show the alignment of ECM. In normal urethra and well repaired urethra, the ECM possesses a direction, but there is no direction in fibrosis or scar. The ICG-001 nanoyarn could facilitate a urethral regeneration without fibrosis.

## Conclusion

In this study, we fabricated a dynamic liquid nanoyarn, which mimiced the native tissue matrix morphologically and structurally, and sufficiently allow cell proliferation and tissue regeneration. The urethroplasty in dog model using optimized ICG-001 delivering nanoyarn resulted in fully functional urethra after tissue reconstruction, confirming the efficacy of drug-delivering nanoyarn in treatments of urethral defect *in vivo*. It could potentially serve as an effective clinical application to cure urethral defects in human patients.

## Data Availability Statement

All datasets generated for this study are included in the article/Supplementary Material.

## Ethics Statement

The animal study was reviewed and approved by the Animal Ethics Committee of Shanghai Sixth People’s Hospital, Shanghai, China.

## Author Contributions

KZ and XF: experimental design. KZ, JZ, RY, and YW: experiments. KZ, XF, JZ, RY, YW, and WZ: data analysis. KZ, XF, JZ, XM, and QF: reagents/materials/analysis tools contribution. KZ, XF, XM, and QF: manuscript writing.

## Conflict of Interest

The authors declare that the research was conducted in the absence of any commercial or financial relationships that could be construed as a potential conflict of interest.

## Supplementary Material

FIGURE S1 The cumulative release of the ICG-001 (concentration vs. time) delivered in different scaffolds in 8 weeks *in vitro*. Dynamic liquid electrospinning scaffold is the nanoyarn.
