# Supplementary material for: Effective Reconstruction of Functional Urethra Promoted With ICG-001 Delivery Using Core-Shell Collagen/Poly(Llactide-co-caprolactone) [P(LLA-CL)] Nanoyarn-Based Scaffold: A Study in Dog Model
Source: Front Bioeng Biotechnol. 2020 Jul 10;8:774. doi: 10.3389/fbioe.2020.00774 (PMC7381300; doi:10.3389/fbioe.2020.00774)

**Table 1 The stress, strain and Young’s modulus of various biomaterials (BAMG, conjugated scaffolds and nanoyarn)**

|  | Stress (MPa) | Strain (%) | Young's modulus (Mpa) |
| --- | --- | --- | --- |
| BAMG | 9.52±1.32 | 627±35 | 1.52±0.13 |
| Conjugate (parallel) | 5.85±0.37 | 415±54 | 1.43±0.21 |
| Conjugate (vertical) | 0.46±0.13 | 836±68 | 0.53±0.09 |
| Nanoyarn (parallel) | 4.13±0.29 | 2385±216 | 0.16±0.03 |
| Nanoyarn (vertical) | 1.87±0.13 | 1423±153 | 0.13±0.02 |

**Table 2 The successful rate of urethroplasty with various biomaterials**

|  | Conjugated scaffold（n=4） | | | Nanoyarn(n=6) | | | | ICG-nanoyarn(n=6) | | | |  |
| --- | --- | --- | --- | --- | --- | --- | --- | --- | --- | --- | --- | --- |
|  | Leakage | Stricture | Success | | Leakage | Stricture | Success | | Leakage | Stricture | Success | |
| 6 weeks | 4 | 4 | 0 | | 1 | 0 | 5 | | 1 | 0 | 5 | |
| 12weeks | 4 | 4 | 0 | | 1 | 2 | 3 | | 1 | 0 | 5 | |
| Success rate |  | 0% |  | |  | 50% |  | |  | 83% |  | |

**Supplementary Figure 1: The cumulative release of the ICG-001 (concentration vs time) delivered in different scaffolds in 8 weeks in vitro. Dynamic liquid electrospinning scaffold is the nanoyarn.**


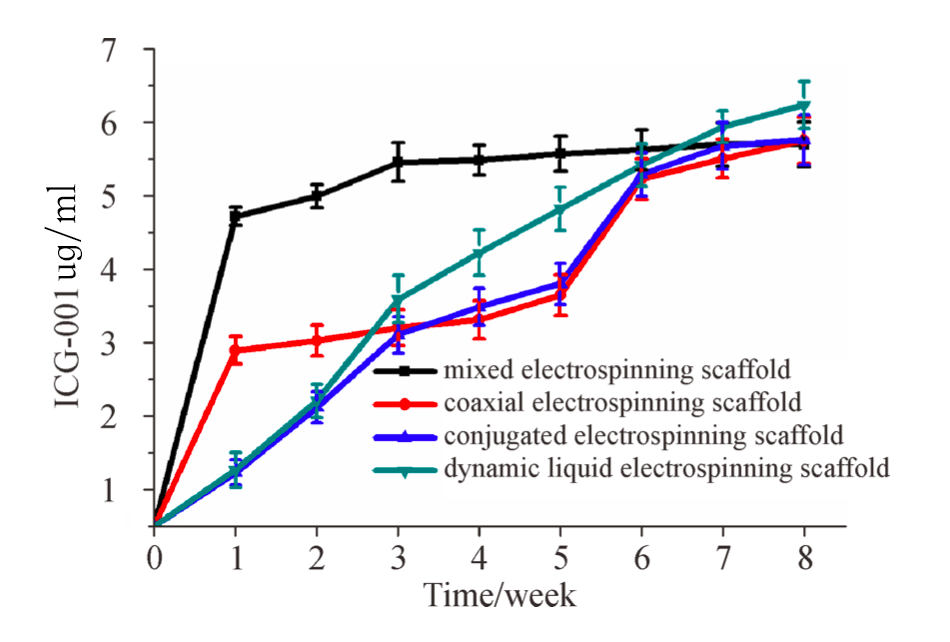

Supplement: FIGURE S1 — The cumulative release of the ICG-001 (concentration vs. time) delivered in different scaffolds in 8 weeks in vitro. Dynamic liquid electrospinning scaffold is the nanoyarn. [file Table_1.DOCX]
